# Hagit Shatkay-Reshef 1965–2022

**DOI:** 10.1093/bioadv/vbac012

**Published:** 2022-03-04

**Authors:** Cecilia N Arighi

**Affiliations:** Department of Computer and Information Sciences, Ammon-Pinizzotto Biopharmaceutical Innovation Building, Newark, DE 19713, USA

**Figure:**
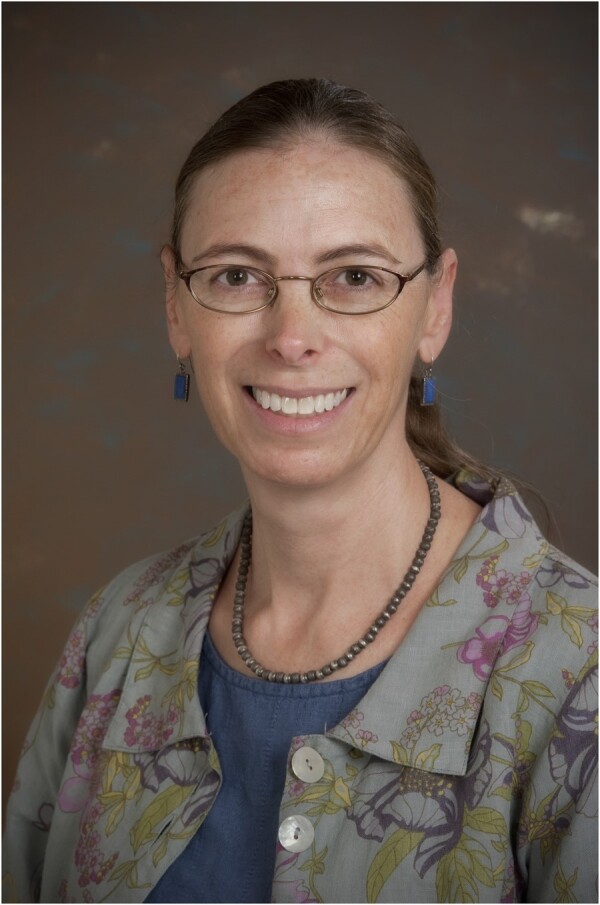
**Professor Hagit Shatkay in 2012** Courtesy of Kathy F. Atkinson/University of Delaware.

Dr Hagit Shatkay, a professor at University of Delaware, passed away on January 2, 2022 in Rockville, MD, USA. This news shocked the scientific community as she had been privately fighting a terminal illness, while giving her best to science. Hagit’s areas of research included machine learning/artificial intelligence, information retrieval and data management and mining with applications in biology, biomedicine and physics. Dr Cathy Wu (Director of the Center for Bioinformatics and Computational Biology and of the Data Science Institute at University of Delaware) introduced me to Hagit when she was appointed at University of Delaware in 2010. As a biocurator, I was interested in finding methods to improve the efficiency of literature curation, and I was amazed by her work, especially in the image and text-based document classification, which was novel and a natural fit to my area of interest. I was very lucky to work with her and Drs Kambhamettu (University of Delaware) and Marai (University of Illinois Chicago), as well as colleagues from MGI and Wormbase databases, in a project aimed at incorporating image-based features into biomedical document classification. Not only was this a very fruitful research experience (Jiang *et al.*, 2019; Li *et al.*, 2021; Trabucco *et al.*, 2021), but also a great opportunity to work with an outstanding researcher, who was very rigorous, and had strong work ethics. This sentiment was echoed by PI Dr Marai, ‘I met Hagit when I was a graduate student, and we became friends. A licensed Cessna plane pilot, she had a reputation of being able to fly, both in her personal life and in science, whereas the rest of us could only walk or drive. She was such a wonderful, complex, modest person and such a strong, thorough researcher and awesome collaborator, who brought light and left light behind her’.

Hagit was born in Israel, where she studied at The Hebrew University, in Jerusalem, earning both a bachelor and a master’s degrees in computer science (*Summa cum laude*). Her master thesis focused on object-oriented databases under advisor Prof. Catriel Be'eri. Then she moved to the USA to conduct a PhD at Brown University, Providence, RI, under Prof. Leslie Pack Kaelbling’s supervision (now a professor at the Massachusetts Institute of Technology). The thesis covered topics such as machine learning, probabilistic models and robotics. Subsequently, Hagit’s interest broadened to medical informatics and thus, in 1999, she was appointed as an IRTA postdoctoral fellow to work on problems related to information retrieval of the literature at the National Center for Biotechnology Information (NCBI). During this time, she developed innovative tools and pioneered applying text-mining methods to improve gene function and large-scale expression experiments (Shatkay and Wilbur, 2000; Shatkay *et al.*, 2000). This led to continuing future collaboration with Dr Wilbur’s group on biomedical text annotation and document classification (Rzhetsky *et al.*, 2009; Shatkay *et al.*, 2008; Wilbur *et al.*, 2006). ‘Hagit was a person who was bright and hardworking and distinguished herself in the field of bioinformatics and her passing is a real loss to the field’, commented Dr Wilbur. In 2001, she moved to work in the private sector and joined the Informatics Research Group at Celera Genomics (Applied Biosystems), where she helped develop algorithms and methods for evaluating whole-genome alignments, improving gene finding and mining the biomedical literature for gene-related information (Istrail *et al.*, 2004). Hagit returned to academia, in 2004, as an assistant professor and Head of the Computational Biology and Machine Learning Lab at the School of Computing at Queen's University (Kingston, Ontario, Canada), and later became associate professor. In 2010, she started a new position, as associate professor at the Department of Computer and Information Sciences, and the Center for Bioinformatics and Computational Biology, at the University of Delaware (Newark, DE, USA), where she later became full professor and the director of the computational biomedicine and machine learning lab in 2018, while maintaining the adjunct professor appointment at Queen’s University.

Hagit’s expertise made her a ‘go to person’ for tackling a variety of real-life problems. For example, developing methods for prediction of protein localization of eukaryotic proteins which integrated several types of sequence and text-based features in collaboration with Dr Kohlbacher’s lab (University of Tübingen, Germany) (Briesemeister *et al.*, 2009; Höglund *et al.*, 2006; Shatkay *et al.*, 2007; Simha *et al.*, 2015); applying machine learning to improve the information that can be gleaned from ‘fuzzy’ EKG and echocardiogram images to help predict which hypertrophic cardiomyopathy patients would develop arrhythmias (Bhattacharya *et al.*, 2019, 2021; Rahman *et al.*, 2014, 2015) in collaboration with Dr Maria Roselle Abraham, MD (Johns Hopkins Medicine Heart and Vascular Institute); and more recently, developing computational methods to analyze data collected through noisy sensors to accelerate data-intensive discovery in astro-particle physics (Stewart, 2019), in an NSF funded project codirected with Dr Christopher Tunnell (assistant professor of physics, astronomy and computer science at Rice University) and Dr Waheed Bajwa (associate professor of electrical and computer engineering and of statistics at Rutgers University).

Hagit’s service to the research community was outstanding. She was an active and insightful member of many scientific societies, including the International Society for Computational Biology (ISCB, senior member), the Association for the Advancement of Artificial Intelligence (AAAI), Association for Computing Machinery (ACM), the American Medical Informatics Association (AMIA) and the International Society for Biocuration (ISB). She served in numerous NIH review panels, in journal editorial boards, and as reviewer of prestigious journals. Most importantly, she enthusiastically participated in several leadership roles in international committees (areas of computational biology, text mining and artificial intelligence) and conferences. For example, she served in the board of directors of the ISCB from 2017 until her passing and was member of the advisory board for BioASQ challenge on large-scale biomedical semantic indexing and question answering from 2013 until her passing.

Hagit was an active participant in multiple important community-driven challenges aiming to develop state-of-the art methods in the field. A few examples include participation in: (i) KDD challenge cup on 2002 on data mining and discovery, where her team got first place in literature mining tasks (Shatkay *et al.*, 2006; Yeh *et al.*, 2003). Dr Hirschman indicated ‘My first contact with Hagit was her entry to a KDD Challenge Cup that my colleague Alex Yeh and I ran in 2002, together with Bill Gelbart’s Flybase group at Harvard. This was one of the very early evaluations to assess ability of text mining to help biocurators. Hagit was one of the developers of the top-scoring system, based on using figure captions to identify key genes in the paper’. (ii) The first large-scale community-based critical assessment of protein function annotation experiment, in which her team included text-based features from the literature for gene functional prediction (Radivojac *et al.*, 2013; Wong and Shatkay, 2013). (iii) the TREC 2005 Genomics track on document classification, applying a thematic probabilistic clustering method with her team at Queen’s University (Zheng *et al.*, 2005). (iv) BioCreative III in tasks related to classification of protein–protein interaction articles teaming up with Dr Luis Rocha (Indiana University, IN, USA) and Dr Anália Lourenço (University of Minho, Braga, Portugal) (Krallinger *et al.*, 2011; Lourenço *et al.*, 2011).

**Figure:**
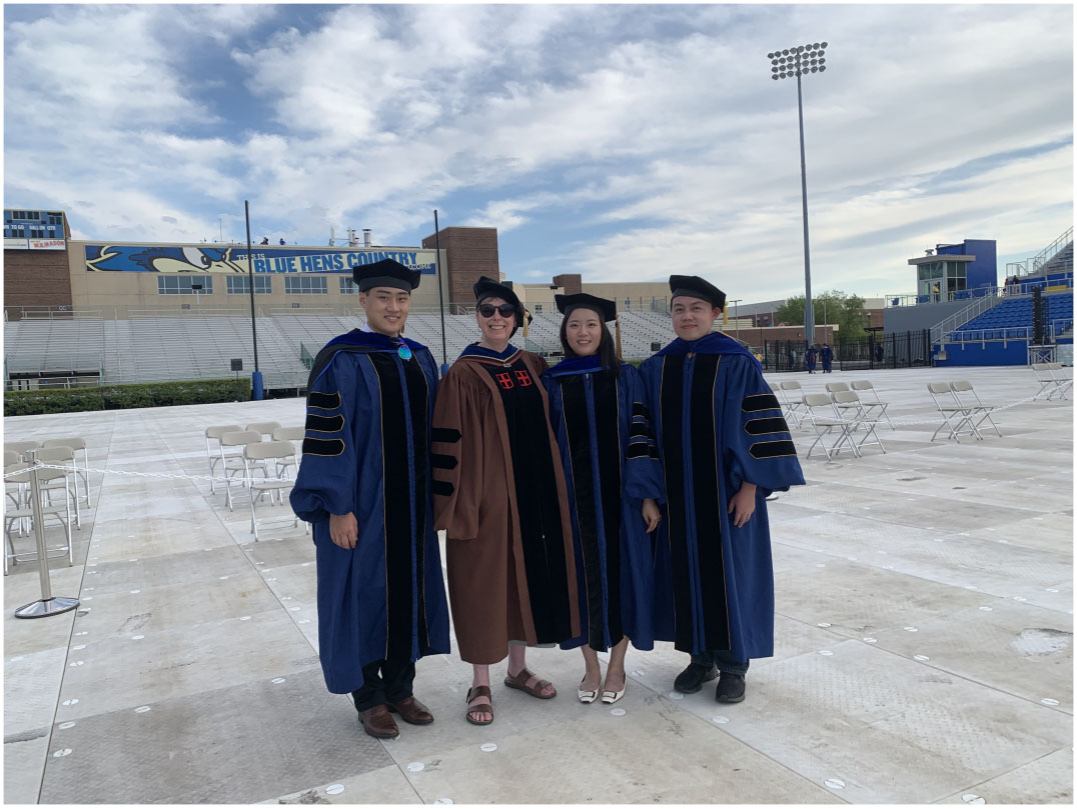
**Dr. Shatkay and students at the 2021** commencement at University of Delaware. Left to right: Gongbo Zhang, Hagit Shatkay, Xiangying Jiang and Pengyuan Li. Courtesy of Gongbo Zhang.

Hagit was a devoted teacher and mentor. While at Queen’s University she established a very strong educational program. In fact, she was awarded the 2005–2006 Howard Staveley Teaching Award, which is given annually to a single faculty member and it is selected by the students at Queen’s School of Computing. Dr Hirschman (MITRE corporation) commented, ‘Hagit was commuting between Rockville, Maryland, and Kingston, Ontario. She would regularly attend teleconferences while commuting—I remember being in awe of how she could do all that driving, keep up with her external engagements like ISMB and BioLINK—and develop a thriving program at Queen’s University’. She was also nominated multiple times for teaching awards while at University of Delaware, and her computational biology course was featured in a UDaily article (Stopyra, 2020) as she integrated real-time analysis of COVID-19 data that enriched student’s experience. She mentored many master and doctoral students. Dr Gongbo Zhang, a former student, highlighted ‘Dr Shatkay was not only my PhD advisor, but also a sincere friend. She pulled me through challenges that seemed impossible to overcome. Her encouragement, “You are capable, and we need to get you stretched,” was one of the most powerful motivations in those difficult times’. Dr Xiangying Jiang, who was mentored by Dr Shatkay during 2014–2020, indicated ‘Dr Shatkay taught me how to think and solve problems during my PhD using her expertise, she also showed me how to face difficulties in work and life using a strong mind, and let me know I should not be ashamed to cry when I fail, but I need to learn from what defeated me’. Dr Pengyuan Li, a former student, highlighted ‘Her passion and dedication to research have inspired me to always look for great challenges. Her patience and detailed feedback carried me carefully through all obstacles and bumps. She was the best advisor!’

I only scratched the tip of the iceberg in terms of describing Hagit’s contributions and collaborations. Her legacy is tremendously rich and she will be deeply missed.
